# Volkmann’s contracture of the forearm due to an insect bite: a case report and review of the literature

**DOI:** 10.1308/003588413X13511609955210

**Published:** 2013-03

**Authors:** J Hardwicke, S Srivastava

**Affiliations:** University Hospitals of Coventry and Warwickshire NHS Trust,UK

**Keywords:** Compartment syndrome, Upper limb, Forearm, Insect bite

## Abstract

Compartment syndrome affecting the upper limb is reported rarely in the literature and is usually limited to single case reports. Upper limb compartment syndrome secondary to envenomation is rare, especially in the UK. Worldwide, it has been reported resulting from snake and insect bites, mostly from snakes from the Viperidae family, and from insects such as bees and wasps. Reports from the UK are limited to one case of an adder bite.

We present a case of a previously fit and well adult who developed an ischaemic contracture of the forearm after an insect bite. Surgical exploration revealed segmental necrosis and contracture of the superficial and deep flexors of the fingers, requiring fasciotomy and tendon-lengthening procedures. This is the first report of a compartment syndrome, or a late ischaemic contracture from an insect bite in the UK. Owing to the rarity of compartment syndrome of the upper limb secondary to envenomation, a delay in diagnosis and treatment can lead to irreversible changes in the muscular compartments of the forearm.

Compartment syndrome affecting the upper limb is reported rarely in the literature and is usually limited to single case reports.^1^ The most common causation is trauma, accounting for nearly 50% of reported upper limb compartment syndromes.^1,2^ Upper limb compartment syndrome secondary to envenomation is rare, especially in the UK. Worldwide, it has been reported resulting from snake and insect bites, mostly from snakes from the Viperidae family and, occasionally, from insects such as bees and wasps.^3^ Reports from the UK are limited to one case of an adder bite causing a localised compartment syndrome in the hand.^4^

Although insect bites are far more common, life or limb-threatening sequelae are rare when excluding anaphylaxis. There are no reports to date of compartment syndrome or secondary ischaemic contracture of the forearm resulting from an insect bite. We present a case of a previously fit and well young adult who developed an ischaemic contracture of the forearm after an insect bite.

## Case history

**Figure 1:**
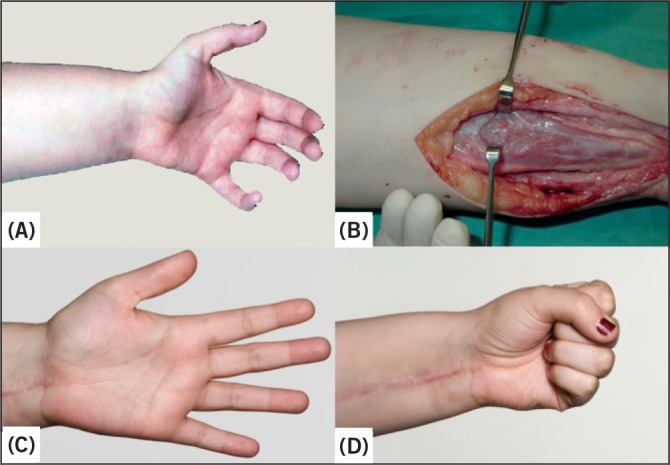
Photographs of the patient’s hand and arm: flexion contracture of the left forearm leading to a ‘clawing’ posture of the hand (A), intraoperative photograph of the muscle belly of the flexor digitorum superficialis showing areas of scarring (B), post-operative photograph at three months showing full extension of all fingers, with no evidence of extensor lag (C) and post-operative photograph at three months showing full flexion of all fingers, with no evidence of overlengthening (D)

A 20-year-old woman presented to our service with a flexion contracture of the non-dominant left hand 6 months after sustaining a bite from a flying insect to the left forearm. The injury was not deemed severe by the patient at the time and initial swelling of the arm was treated with antihistamines. No dressings or constrictive bandages were applied. The sustained swelling of the left forearm and development of the flexion contracture of the hand (Fig 1a) led her to seek medical advice. Haematological and biochemical analysis as well as autoimmune screening at this time were normal, as were nerve conduction studies. Magnetic resonance imaging showed oedema of the deep and superficial flexor compartments of the forearm. A diagnosis of Volkmann’s contracture was made (Holden type I mild).

Surgical exploration was performed under tourniquet control and was approached via a volar linear incision from mid-forearm to mid-palm to allow access to the carpal tunnel. Areas of necrosis were noted in the flexor digitorum superficialis (FDS) muscle (Fig 1b) and muscle biopsy was taken from the muscle belly as well as the musculotendinous junction. Contracture of both the FDS and the flexor digitorum profundus (FDP) was noted. A volar compartment fasciotomy was performed and the carpal tunnel released. Tendon lengthening of the FDS to index, middle and ring fingers, and the FDP to middle, ring and little fingers was completed with sacrifice of the FDS to the little finger. The transverse carpal ligament was lengthened and repaired to prevent bow stringing.

The repairs were supported in a dorsal splint and hand therapy commenced the following day with a controlled active movement regimen. At clinical review after three months, full range of movement was present (Figs 1c and 1d) as well as normal sensibility. Histological analysis revealed a localised vasculitis with progressive fibrosis, with a florid chronic inflammatory cell perivascular cuff.

## Discussion

Compartment syndrome exists when the pressure in an osteofascial envelope rises to a level that impairs cellular function and, when sustained, this can lead to irreversible changes in the contents of that compartment.^2^ Volkmann, to whom the long-term sequelae of raised intracompartment pressures is credited, described his clinical findings in 1881 with the observation of a rapid-onset paralysis and contracture in tightly bandaged limbs, attributed previously to nerve compression rather than, as he suspected, muscle compression and necrosis.^5^ Volkmann observed these findings more commonly in the upper limb than the lower limb.

This is the first report of a compartment syndrome or a late ischaemic contracture of the forearm from an insect bite. Previous reports from the worldwide literature are limited to a localised compartment syndrome of the hand. Insects such as wasps and bees produce a wide variety of toxins such as amines, peptides and enzymes that can cause local and systemic inflammatory reactions after a sting.^3^

Owing to the rarity of compartment syndrome of the upper limb secondary to insect or snake bite, a delay in diagnosis and treatment can lead to irreversible changes in the muscular compartments of the forearm, as was shown in this case. In the UK literature, Saravanan *et al* reported a transient clawing of the hand after a bee sting but compartment syndrome was not diagnosed and symptoms resolved without surgical intervention.^6^ Tucker and Josty reported compartment syndrome of the thumb and thenar eminence from an adder bite, requiring surgical decompression.^3^ Cawrse *et al* reported lower limb compartment syndrome from adder bite, again, requiring a fasciotomy.^7^

In our reported case, no other traumatic cause was elicited from the medical history, no biochemically detectable systemic illness was recorded and histological analysis of the muscle refuted the diagnosis of underlying intrinsic inflammatory muscle pathology. A localised vasculitis with progressive muscle fibrosis of unknown origin was diagnosed. We must assume this was secondary to the insect bite. Owing to the absence of classical clinical pointers to compartment syndrome, early intervention was missed, leading to the development of contracture. A more severe type of contracture would require either a muscle slide procedure or tendon transfer, increasing postoperative morbidity, as well as the treatment of associated nerve injury.

The senior author’s management plan to release the compartment and carpal tunnel at the time of surgical exploration, combined with the lengthening of both the involved flexor tendons and carpal tunnel, led to a satisfactory outcome and full return to the premorbid range of motion and function.
